# What group 2 innate lymphoid cells tell themselves: autocrine signals play essential roles in mucosal immunity

**DOI:** 10.1038/s41392-021-00685-2

**Published:** 2021-07-09

**Authors:** Yang Zhao, Wenxian Guan, Xiaofei Shen

**Affiliations:** 1 State Key Laboratory of Membrane Biology, Institute of Zoology, Chinese Academy of Sciences, Beijing, China; 2 Department of General Surgery, Nanjing Drum Tower Hospital, Affiliated Hospital of Nanjing University Medical School, Nanjing, Jiangsu Province China

**Keywords:** Innate immune cells, Inflammation

Recently, two article papers (Chu et al.^1^ and Roberts et al.^2^) were published describing that autocrine regulation mediated by the choline acetyltransferase (ChAT)-acetylcholine (ACh) is critical for optimal Group 2 innate lymphoid cell (ILC2) responses during helminth infection and allergen challenge.

ILC2s are tissue-resident lymphocytes enriched at barrier surfaces, acting as important regulators of mucosal immunity. However, it is largely unknown how ILC2s can instruct proper immune responses to pathogenic signals. Autocrine regulation is an important form of signaling activity within the nervous system, with the advantages in enabling tight coupling of synaptic activity to signal transduction, and also in sustaining or amplifying intercellular paracrine cues. As emerging evidence have shown a complex crosstalk between nervous system and ILC2s, is it possible that the autocrine regulation mode in neuron cells also applies to ILC2 responses?

The numbers of mucosal ILC2s are low, and immediate sense of danger signals from invading pathogens as well as the following appropriate effector responses should be raised in ILC2s to compensate for the delayed immunity from adaptive T cells. The anatomical co-localization nature of neurons with ILC2s has enabled them to be capable of doing such a pivotal “job,” allowing signaling molecules of the the nervous system such as neuropeptides and neurotransmitters to function on ILC2s with a quickly amplified responses.^3^ As the first neurotransmitter discovered, roles of ACh are largely discussed in the nervous system. In the context of immune responses, ACh is best known for its cholinergic anti-inflammatory signaling mediated mainly by α7 nicotinic acetylcholine receptors (nAChR) on target cells. ILC2s express α7nAChR and selective agonist of this receptor reduced ILC2 effector function.^4^ Parasitic helminths such as *Nippostrongylus brasiliensis* release secreted forms of active acetylcholinesterase into their host environment, which are postulated to act to dampen host cholinergic responses. Does this indicate that protective immunity against helminth infection raised by ILC2s requires ACh signaling?

To address this question, both groups utilized ChAT^BAC^-eGFP mice and found that ILC2s synthesized and released ACh early after infection with *N. brasiliensis*. ChAT-eGFP expression was markedly upregulated only in ILC2s among the leukocyte populations screened. With the use of CD25 as surface marker to distinguish natural ILC2s (nILC2s) and inflammatory ILC2s (iILC2s), Chu et al.^1^ found that most ChAT^+^ ILC2 were CD25^−^ cells, indicating a potential inflammatory phenotype of these ChAT^+^ ILC2s. Robert et al.^2^ further extended out understanding in these cholinergic ILC2s with the use of multiple surface markers. They observed that ChAT^+^ ILC2s displayed a range of phenotypes for both nILC2s and iILC2s with the most important association with ChAT expression being related to a more activated-like profile of ILC2s rather than associated with a specific ILC2 subset. The cholinergic phenotype in ILC2s was a general feature of type 2 immunity and alarmin signaling pathways including interleukin (IL)-33 and IL-25 potently induced these ChAT-eGFP-expressing ILC2s both in vivo and in vitro. With the use of different genetic strategies to disrupt ChAT expression in ILC2s, both groups found a biological significance of ACh production derived from ILC2s during helminth infection. Whether ACh in ILC2s functions in an autocrine way or paracrine way on other types of cells? Both groups found that ILC2s expressed a wide variety of ACh receptors other than the identified α7nAChR after activation. ILC2-derived ACh promoted autocrine population expansion of ILC2s, whereas its role in ILC2 cytokine production requires further investigation. Chu et al.^1^ found that ILC2-derived ACh played a role in promoting type 2 cytokine expression by ILC2s, whereas Roberts et al.^2^ found that ILC2s genetically unable to synthesize ACh did not have a reduced production of IL-5 and IL-13 on a cell-by-cell basis. The overall decreased numbers of IL-13^+^ and IL-5^+^ ILC2s in the study by Robert et al.^2^ were linked to fewer ILC2s at baseline and a diminished expansion of ILC2 during infection. Blockade of ACh receptors can significantly diminish ILC2 responses, although different antagonists were used in these two studies: Robert et al.^2^ found that muscarinic acetylcholine receptors (mAChR) blockade, but not nAChR blockade, inhibited ILC2 proliferative response in vitro, whereas Chu et al.^1^ found that both mAChR and nAChR blockade inhibited IL-13 production by ILC2s. Taken together, these results suggest that ILC2s can produce molecules typically made by the nervous system, such as neurotransmitters, to regulate their own proliferation and function in an autocrine way.

Autocrine regulation mediated by ACh in ILC2s is also important for the maintenance of ILC2s. Lower numbers of ILC2s in uninfected *Chat*-deficient mice were identified, suggesting a homeostatic requirement for ACh. IL-2, which is critical for ILC2 maintenance, can also induce ChAT expression by itself. Furthermore, ChAT expression in ILC2s was maintained even after helminth eradication, which raised a possibility that autocrine regulation of ILC2 responses by neurotransmitters may have a profound effect during the later stage of infection. How ACh regulates the pool of ILC2s under homeostasis and infection is still unknown, which may both include a direct effect of ACh on ILC2s proliferation or the effects on surface receptors such as ST2 and ICOS.

**Fig. 1:**
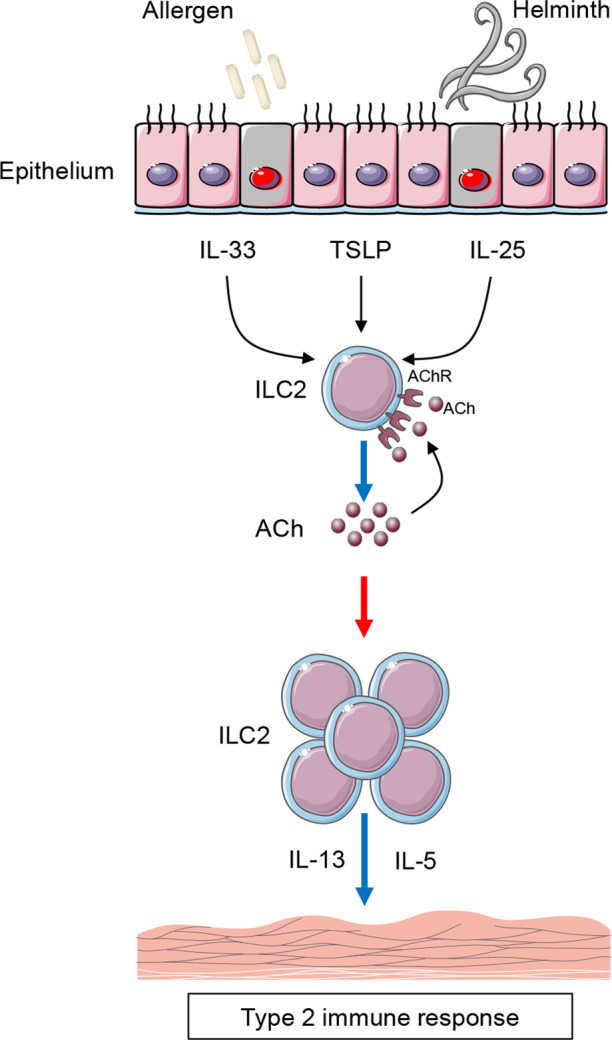
Autocrine regulation of ILC2 responses mediated by ChAT-ACh pathway during helminth infection and allergen challenge. ILC2s upregulate choline acetyltransferase (ChAT)—the enzyme responsible for the biosynthesis of acetylcholine (ACh) after helminth infection or challenging with allergens or alarmin cytokines including IL-33, IL-25, and thymic stromal lymphopoietin (TSLP). ILC2s also express numerous acetylcholine receptors, which can respond to ACh derived from ILC2s to form an autocrine feedback loop. Autocrine regulation of ILC2 responses mediated by ChAT-ACh pathway is indispensable for ILC2 effector function through manipulating their proliferation and cytokine production such as IL-13 and IL-5

In general, autocrine regulation mediated by neurotransmitters such as ACh builds a bridge between the nervous system and immune system, which helps to explain why ILC2s can exert their function immediately after pathogen insult, and raises a question that whether ILC2s can respond to neurotransmitters to regulate the release of ACh other than well-known alarmin signals. In addition, it is interesting to further elucidate whether neurotransmitters produced by ILC2s can also function on the nervous system in a paracrine way. Current studies clearly suggest that different neuropeptides and/or neurotransmitters may lead to divergent outcomes in ILC2s. Therefore, a more comprehensive understanding in the molecular control of ILC2 function is also urgently needed, which will benefit future development of targeted precision therapies (Fig. 1).
